# Long- term effects of previous experience determine nutrient discrimination abilities in birds

**DOI:** 10.1186/1742-9994-5-4

**Published:** 2008-02-22

**Authors:** H Martin Schaefer, Kathrin Spitzer, Franz Bairlein

**Affiliations:** 1 Faculty of Biology, University of Freiburg, Department of Ecology and Evolutionary Biology, Hauptstr. 1, 79104 Freiburg, Germany; 2 Institute of Avian Research, 'Vogelwarte Helgoland', An der Vogelwarte 21, 26286 Wilhelmshaven, Germany

## Abstract

**Background:**

Foraging behaviour is an essential ecological process linking different trophic levels. A central assumption of foraging theory is that food selection maximises the fitness of the consumer. It remains unknown, however, whether animals use innate or learned behaviour to discriminate food rewards. While many studies demonstrated that previous experience is a strong determinant of complex food choices such as diet mixing, the response to simple nutritional stimuli, such as sugar concentrations, is often believed to be innate.

**Results:**

Here we show that previous experience determines the ability to track changes in sugar composition in same-aged individuals of a short-lived migratory songbird, the garden warbler (*Sylvia borin*). Although birds received identical foods for seven months prior to the experiment, wild-caught birds achieved higher sugar intake rates than hand-raised birds when confronted with alternative, differently coloured, novel food types. Hand-raised and wild birds did not differ in their initial colour selection or overall food intake, but wild birds were quicker to adjust food choice to varying sugar intake.

**Conclusion:**

Over a period of at least seven months, broader previous experience translates into a higher plasticity of food choice leading to higher nutrient intake. Our results thus highlight the need to address previous long-term experience in foraging experiments. Furthermore, they show that hand-raised animals are often poor surrogates for testing the foraging behaviour of wild animals.

## Background

Diet selection is typically viewed as an adaptive process that aims to maximize fitness of the consumer [1]. Central to this adaptive theory is that animals associate food with their fitness consequences, but, owing to the delay between consumption and postingestive consequences, the mechanisms for such an association are poorly resolved [2]. As a consequence, it is not well understood how the evolutionary history of a species, the physiological state of an individual animal, and its previous experience interact in shaping foraging behaviour [e.g., [3,4]]. In particular, it remains contentious whether food selection in animals is largely a function of learning or of genetically determined, innate behaviour [5-7].

According to the literature, the relative importance of learning and innate behaviour in foraging decisions of vertebrates seems to be context dependent. Innate behaviour is particularly important as a response to colour stimuli, when it serves to avoid harmful prey at first encounter, such as poisonous snakes or insects [8,9]. It is likewise important for identifying food resources such as fruits at early age [10,11]. In contrast, learning is fundamental for the formation of ecological niches translating into habitat and foraging preferences [12]. As a consequence, learning may lead to dietary conservatism causing food choice to be a reflection of the environmental conditions experienced at early age [5,13]. Dietary conservatism is not only reliant on cultural transmission of feeding habits, but also on individual experience as this phenomenon is not restricted to social foragers, occurring more widely in birds, herbivorous mammals and insects as well as fish larvae [3,13-16]. However, it has been argued that dietary conservatism might be maladaptive in omnivores that feed on a variety of foods, particularly in environments where food abundances might shift unpredictably [7].

A prerequisite for adaptive food selection is to discriminate prey according to its nutrient contents. In many species, nutrient discrimination abilities are well developed, particularly for differences in sugar contents because these can be detected based on gustatory cues [17-19]. Although nutrient discrimination abilities contribute to the plasticity of food choice, and impaired discrimination abilities might be responsible for the development of dietary conservatism, the relative importance of innate behaviour and learning for the development of nutrient discrimination is as yet unknown. In particular, no study assessed whether nutrient discrimination abilities are dependent on previous experience and whether such effects persist for prolonged time periods.

If there is a learned component of nutrient discrimination, we predicted that those individuals, which had previously experienced a broad range of prey items, will have higher nutrient intake than individuals that were fed invariant food. We document how previous experience has long-lasting effects (i.e., more than seven months) on the nutrient discrimination abilities of a short-lived, omnivorous bird, the garden warbler (*Sylvia borin*). In particular, we show that broader previous experience leads to higher plasticity in food choice when encountering novel food types. Experienced birds were quicker to adjust their food intake to varying nutritional contents, thereby achieving a higher nutrient intake.

### Study design

To test whether nutrient discrimination abilities depend on a learned component, we compared food choice in same-aged hand-raised and wild-caught garden warblers. We analysed food choice during the migration period because this is the time when birds are under increased selective pressure for optimal nutrient intake [20,21]. In order to assess whether previous experience has long-lasting effects on nutrient selection, both groups received identical foods for a period of seven months prior to the experiments. This design allowed us to exclude well-known biases caused by pre-conditioning animals on a particular food. Under the hypothesis that nutrient discrimination abilities are learned, we predicted that wild-caught birds exhibit higher plasticity in food choice and better discrimination abilities because they were previously exposed to higher variability in prey items and prey quality. Under the hypothesis that adaptive food choice is an innate behaviour both groups should exhibit similar nutrient discrimination abilities when encountering novel food. In a paired design, we used alternative, novel food items, which differed in colour, texture, and nutritional composition from known food, to test nutrient discrimination rather than the tendency to consume familiar food, i.e., dietary conservatism. Alternative foods had different colours as conditional stimuli, which were alternated between experiments. Our design thus compares how learning and innate behaviour contributes to food choice based on visual and nutritional stimuli in a novel context.

## Results

**Figure 1:**
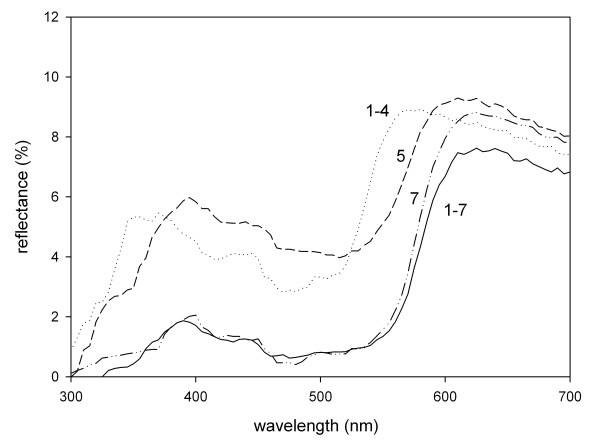
**Reflectance spectra of artificial foods.** Numbers in the panel identify the number of the experiments. The dotted line indicates the reflectance of orange foods, the solid line that of red cubes. Note the minor difference between reflectance spectra used in experiments 5 and 7. Overall reflectance is low as agar cubes partly transmitted the light that illuminates their surface.

**Figure 2:**
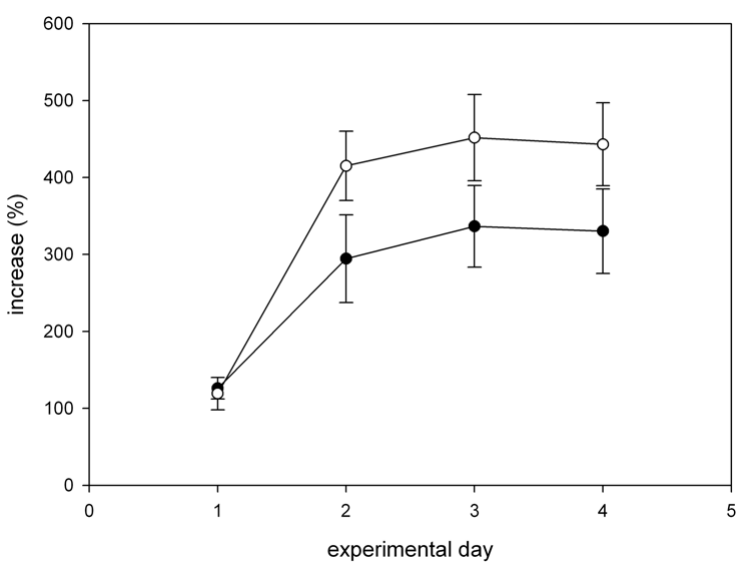
**Wild-caught birds (white dots) increased the intake of the sugar-rich food more quickly and more strongly than hand-raised birds (black dots) during the experiments.** We illustrated this difference for food intake in trial two. Note that the food intake of both groups reached a plateau on the second day. Illustrated are means and standard errors.

**Figure 3:**
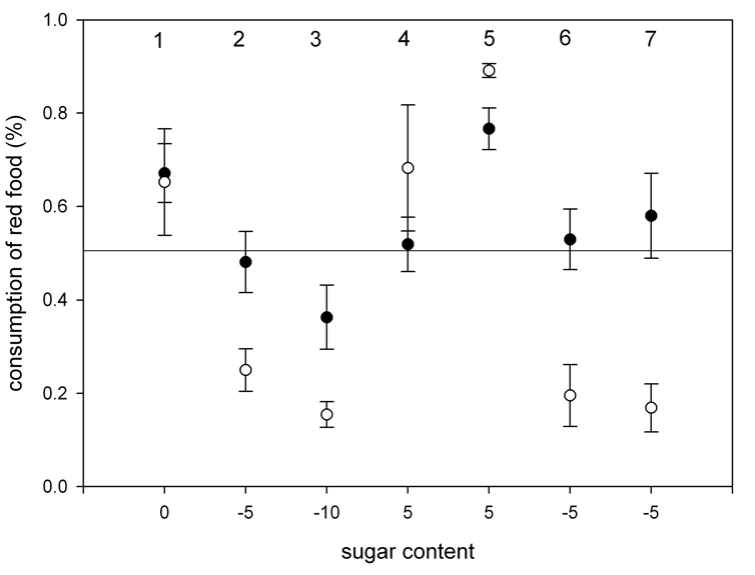
**Food intake of wild-caught (white dots) and hand-raised (black dots) birds; illustrated are means and standard errors.** Numbers in the upper part of the panel identify individual experiments. Values on the x-axis identify the difference in sugar contents between both foods (in % of overall weight). Positive values indicate that red foods contained more sugar than orange foods, whereas negative values indicate higher sugar contents in orange foods. The line indicates food intake if birds consumed both foods equally.

**Figure 4:**
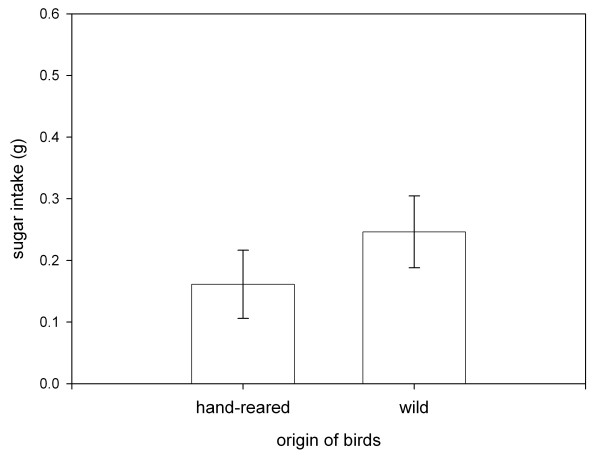
Wild-caught birds had higher overall sugar intake in the experiments than hand-raised birds. Illustrated are means and standard errors.

When offered a choice between two differently coloured artificial fruits of identical nutritional composition, birds consistently consumed more red than orange fruits (t-test, df = 17, t = 2.99, p < 0.01). Wild-caught and hand-raised birds did not differ in their initial preference for red (t-test, df = 17, t = 0.13, p > 0.8). In the following six experiments, which all lasted four consecutive days, we manipulated sugar contents to test whether birds tracked changes in nutrient contents. After every second experiment, we alternated the sugar contents of red and orange fruits (see table 1) and reduced the colour difference in the last three experiments (Fig. 1). The duration of the experiments allowed birds to adjust their food intake as the intake of both groups reached a plateau, but the learning curve differed predictably among groups (Fig 2). We found that wild-caught birds tracked changes in food quality more readily than hand-raised birds (repeated measurement GLM, between subjects factor: birds' origin F= 9.64, df = 1, p < 0.01, no significant within-subject factors). Food selection of hand-raised and wild-caught birds differed in all experiments except for experiment 4, but particularly when colour differences were reduced (Fig. 3). Overall, both groups had equal variance in food intake (in all but experiment 5, Levene test for unequal variances, df_1,17 _p > 0.1), and the total food intake did not differ between groups (repeated measurement GLM p > 0.05). Yet, the better nutrient discrimination of wild bird resulted in higher sugar intake (repeated measurement GLM, between subject factors: birds' origin F= 6.3, df = 1, p < 0.05, no significant within-subject factors; Fig. 4).

**Table 1:** Glucose contents of red and orange agar cubes used during the experiments.

	Foods		
	red	orange/light red	Contrast (jnds)
Trial 1	10%	10%	56.1
Trial 2	4%	9%	56.1
Trial 3	4%	14%	56.1
Trial 4	9%	4%	56.1
Trial 5	9%	4%	36.6
Trial 6	4%	9%	23.6
Trial 7	4%	9%	14.2

For each experiment, the contrasts between both colours are given in jnds (see methods), lower values represent lower colour contrasts.

## Discussion

Wild-caught garden warblers showed better nutrient discrimination abilities than hand-raised birds if confronted with varying sugar contents and diminishing colour differences in serial presentation of two alternative foods. Before discussing this result, we address potential biases of our study design. The small sample size of wild-caught birds might bias our results. We consider this possibility unlikely because low sample size restricts the power of statistical tests, thus reducing the likelihood of finding consistent differences. Even more importantly, both groups behaved similarly in the experiments without differing in overall food intake, in the variability of food selection, in time needed to adjust their food intake over the course of the experiments (Fig. 2), or in initial colour preference. Finally, the discrimination abilities of wild-caught garden warblers are similar to those reported for that species or for wild-caught birds of other species which already discriminate 1–2% differences in sugar contents [18,19,22]. Another potential bias would be if hand-raised birds had shown a higher degree of dietary conservatism preferring the food most similar to their maintenance food prior to the experiments. However, hand-raised birds significantly increased their intake of sugar-rich food during experiments 2 and 3, documenting that they indeed preferred higher sugar contents than those they were accustomed to. Thus, artificial rearing conditions did not necessarily lead to a higher degree of dietary conservatism. However, although birds that were raised on the synthetic food had seasonal body mass chances similar to wild birds, we cannot rule out that this food was missing some nutrients that might influence diet choices later in life.

### Nutrient discrimination

Our experiments show that previous experience influences nutrient discrimination suggesting an underlying learned component. Wild-caught birds selected always the sugar-rich food, whereas food selection in hand-raised birds departed from a non-random choice only twice. In general, nutrient discrimination might be achieved directly by taste or indirectly by metabolic feedbacks [23,24]. The larger experience of wild-caught birds might thus have led to behavioural or physiological adaptations that allowed them to forage more efficiently among prey of varying quality. Alternatively, foraging theory predicts that foragers perform better in variable environments if they rapidly devaluate past experience [25]. According to this idea, wild-caught birds might be better foragers because they devaluate information on the relationship between colour and sugar contents from preceding experiments more quickly. Regardless of the exact mechanism, we conclude that artificial rearing conditions impaired food discrimination ability in garden warblers. This result has important implications for using laboratory animals (mice, rats, chicken) as model organisms to study food selection. If artificial rearing conditions bias nutrient discrimination abilities, laboratory animals are poor representatives of the behaviour of free-living animals.

Previous experience is an important determinant of food choice as many studies showed that animals prefer prey they are familiar with [e.g., [5,15,16]]. Thus, experience at early age often results in foraging decisions becoming more rigid later in life. In contrast, the novel contribution of our experiments lies in the observation that broader early experience increases the plasticity of foraging decisions when birds encounter novel food later in life. In particular, we show that differences in previous experience have long-lasting effects on food selection resulting in a higher sensitivity to track variation in nutritional contents. Such long-lasting effects represent a functional explication for the pronounced inter-individual variability that often characterise food selection experiments [26,27].

### Visual cues in food selection

Food colour is an important determinant of foraging decisions in many organisms including humans [28,29]. Consistent colour preferences for red are documented in fish and some fruit-eating birds [10,11,30], probably as an adaptation to find nutritious fruits. Our study shows that wild-caught and hand-raised garden warblers – the latter had no previous experience with colour cues other than brown or beige before – both initially selected red over equally nutritious orange food. This result documents that birds possess innate colour preferences for red. Since fruits are predominantly red during the European summer [10,31], these preferences are probably adaptive because they enable birds to find edible fruits, which are an easy prey, particularly for inexperienced, young birds.

Our experiment shows that foraging decisions of wild-caught birds were less influenced by colour because they primarily selected food according to sugar contents. The lower priority that experienced birds gave to visual cues is consistent with the loss of innate colour preferences in the related blackcap [10,32]. We thus suggest that birds learn to prioritize cues related to metabolic feedback or taste over visual cues when selecting food in a variable environment.

## Conclusion

We document long-term effects of previous foraging experience on the nutrient discrimination ability of an omnivorous songbird. Our results demonstrate a learned component even in simple foraging decisions such as those between two simultaneously offered foods that differ in the contents of a single nutrient. The situations that animals face when foraging naturally are highly complex because prey items vary in many dimensions, such as macronutritional, micronutrional, and allelochemical contents. If learning results in more efficient foraging in simple situations, it is likely to be critical for coping with complexity. Adaptive theory assumes that individuals can modify their behaviour to respond optimally to environmental variability. Here we showed that broad previous experience in foraging leads to higher plasticity when birds encounter novel food. This result demonstrates that laboratory animals might be poor surrogates for inferring the behaviour of free-ranging animals.

## Methods

### Bird maintenance

Garden warblers are omnivorous birds that consume insects and fruits, the latter being particularly important prior to and during migration as well as in the winter quarters [33]. All individuals used in the experiment fledged in 2003. Five birds were wild-caught prior to their migration in August 2003 in Wilhelmshaven, Germany (53°3' N, 8°1' E). Further 14 birds originated from seven clutches of unrelated birds held at the Vogelwarte Helgoland. These birds were hand-raised and all birds were fed mealworms (*Tenebrio molitor *larvae) and a beige standard maintenance diet that was specifically developed to meet the nutritional requirements of this species [34] with water available *ad libitum*. The standard maintenance diet consisted (by wet mass) of 14.1% crude protein (casein), 10% crude fat (plant oils), 5% carbohydrates (glucose and fructose), 5% vitamin and mineral mix, 15.9% fibre and 50% water [34]. All birds were held individually in cages 38 × 45 × 24 cm under constant conditions (light : dark 12 : 12; 20 ± 1°C; approx. 60% relative humidity). Birds were regularly weighted and their welfare was monitored. Birds were fed identical food for seven months prior to the experiment. Hand-raised birds had not experienced coloured stimuli other than brown and beige food before the experiment.

### Experimental design

Experimental foods consisted of two agar cubes with a weight of 5 g ± 0.1 g each. Agar, glucose, artificial colour (orange or varying amounts of red; Brauns Heitmann GmbH & Co., Warburg, Germany), cellulose, and water were added to agar cubes (see [35]). Because agar cubes contained considerably less energy than the maintenance diet that birds were used to, we assumed that all birds aimed to maximise their energy intake when consuming agar food. Agar cubes were presented in glass cups at the bottom of the cages. To exclude that food choice was affected by contrasts between cubes and background (see [35]), we placed glass cups on carton paper of the same colour (i.e., orange fruits were presented against an orange background, red fruits against red background). We found no difference between wild-caught and hand-raised birds in the latency to consume agar cubes.

All experiments lasted for four consecutive days, and we analysed the mean food intake over this time span. During the experiments, birds had *ad libitum *access to water but no access to the standard food. Birds did not feed during the night and experiments were run each day for the first 90 min of the light phase, when birds were hungry and foraged actively. To assess whether initial preference differed from food selection after 90 min, when metabolic feedback might be more pronounced, we compared food intake after 15 min and 90 min. There was a strong correlation between both measures (r = 0.79, p < 0.0001) and we report the results after 90 min. At the end of the experiments, we replaced the agar cubes with maintenance food and calculated the amount that individual birds ate of each agar cube. Experiments were run during the spring migration in April and May 2004, when birds were almost a year old. Birds were kept under license of the district government Weser-Ems, Oldenburg, Germany. All trials conform to current laws.

We first tested whether wild-caught birds differed from hand-raised birds in their initial colour preference presenting orange and red agar cubes with identical sugar contents. We then alternated sugar contents after every second experiment (see table 1) to test whether both groups tracked changes in nutritional quality equally well. We conducted these experiments in the same order, rather than randomly, because we reduced the colour difference during the course of our experiments. Thus, the results of a given experiment are not necessarily independent of the results of the preceding experiment, but we were precisely interested in testing whether such carry-over effects differed between groups.

Food selection might depend on the relative difference in nutrient contents of two foods, but it also depends on the absolute sugar contents obtainable in foods. We therefore decreased sugar contents from the second experiment onwards. We used 5% difference in sugar contents because garden warbler likely perceive this difference given that some frugivorous birds already discriminate 1–2% differences in sugar contents [19]. In experiment three, we increased the difference between both foods to 10% because hand-raised birds had not chosen the more rewarding orange food when it contained only 5% more sugar in experiment two. When both groups consumed less red food in experiment three, we offered red cubes with higher sugar contents (experiment 4 & 5) and switched sugar concentrations again in experiment six and seven so that orange cubes contained more sugar. To assess whether diminishing the colour difference between alternative foods constrained food selection, we added red colour to the orange cubes from experiment five on.

### Colour measurements and contrast calculation

The colours of cubes were checked with a diode-array spectrometer (Ocean Optics USB2000). We used a Top Sensor System Deuterium-Halogen DH-2000 lamp as a standardized light source and a coaxial fiber cable (QR400-7, Ocean Optics) that was mounted inside a matt black plastic tube to exclude ambient light. Reflectance was measured as the proportion of a standard white reference tile (diffuse PTFE; WS-2). The angle of illumination and reflection was fixed at 45° to minimize glare. Spectra were processed with SpectraWin 4.0 software and calculated in 5 nm intervals from 300 – 730 nm. The spectra had peak reflectance at longer wavelength but showed a lower secondary peak at wavelength ranging from 350–390 nm (see Figure 1), similar to orange and red carotenoid reflectance spectra.

To calculate food colour according to avian perception, we used an eye model that is based on the spectral sensitivities and the receptor noise of the four cone types that are assumed to function in avian colour discrimination [36]. Based on analytical approximation of cone visual pigments and oil droplet spectra, the model calculates cone excitation values for each spectrum. The chromatic contrasts between the two colours of the agar cubes were calculated as the log of the quotient of quantum catches of photoreceptors from both spectra (see [37] for equations). The chromatic contrasts describe how much the colours of agar cubes are separated in avian colour space. The units for chromatic contrasts are jnds (just noticeable differences), 1 jnd is at the threshold of discrimination, values less than 1 jnd indicate that two colours are indistinguishable, and values above 1 jnd how easily spectra can be discriminated. Data on the spectral sensitivity of garden warblers are not available. Because most passerine birds possess a UVS cone, we used cone sensitivities of another passerine bird with a UVS cone, the blue tit (*Cyanistes caeruleus*) [38].

### Statistical analyses

We tested birds' initial colour preferences with paired t-tests. To test nutrient discrimination abilities, we used repeated measurement generalised models (GLMs) that account for the fact that individual choices are not independent in different experiments. We conducted several repeated measurement GLMs, all had sex and birds' origin as independent factors, but different dependent variables. Sex had no significant effects and was thus excluded from all models. To assess nutrient discrimination abilities, we conducted a repeated measurement GLM with the percentage of red food consumed in each trial as dependent variable. To assess whether groups differed in sugar intake or overall food intake, we ran models with these variables as dependent variable. To assess changes in the food intake between experiments, we calculated the daily food intake in relation to the mean food intake of the preceding experiment. All analyses were done with SPSS 14.0.

## Competing interests

The author(s) declare that they have no competing interests.

## Authors' contributions

HMS, KS and FB designed the research, KS performed the experiments, HMS analysed the data and wrote the manuscript. All authors read and approved the final manuscript.
